# Characterization of chlorophyll binding to LIL3

**DOI:** 10.1371/journal.pone.0192228

**Published:** 2018-02-01

**Authors:** Astrid Elisabeth Mork-Jansson, Lutz Andreas Eichacker

**Affiliations:** Centre for Organelle Research, Faculty of Science and Technology, University of Stavanger, Stavanger, Norway; Arizona State University, UNITED STATES

## Abstract

The light harvesting like protein 3 (LIL 3) from higher plants, has been linked to functions in chlorophyll and tocopherol biosynthesis, photo-protection and chlorophyll transfer. However, the binding of chlorophyll to LIL3 is unclear. We present a reconstitution protocol for chlorophyll binding to LIL3 in DDM micelles. It is shown in the absence of lipids and carotenoids that reconstitution of chlorophyll binding to *in vitro* expressed LIL3 requires pre-incubation of reaction partners at room temperature. We show chlorophyll a but not chlorophyll b binding to LIL3 at a molar ratio of 1:1. Neither dynamic light scattering nor native PAGE, enabled a discrimination between binding of chlorophyll a and/or b to LIL3.

## Introduction

The light harvesting like membrane protein 3 (LIL3) is a member of the light-harvesting complex protein family (LHCP) and characterized by the presence of a common LHC sequence motif [1]. LIL3 is classified in the group of two-helix stress enhanced proteins (SEPs). Common ancestors of SEPs are the high-light inducible proteins (HLIP) in cyanobacteria, and the one-helix proteins (OHP) in plants [2]. LHCP genes harbor two LHC motifs which are associated with Chlorophyll (Chl) binding [1, 3, 4]. However, also proteins that harbor only one LHC motif, like LIL3, were determined to bind Chl. Microscale thermophoresis studies indicated that dimerization could provide the functional compensation for the missing LHC motif [5].

LIL3 was identified as a fluorescent native gel band containing Chl upon the onset of deetiolation in etiolated barley (*Hordeum vulgare*) [6]. In isolated etioplasts, Chl_GG_, Chl_PY_, and very small amounts of Chlorophyllide (Chlide) a were extracted from the fluorescent LIL3 complex upon induction of Chl synthesis. Data showed Chl a binding and indicated that dimerization of LIL3 preceded binding of Chl a [5]. Recently, the binding of Chl a and Chl b to LIL3 was reported using a tryptophane fluorescence quench analysis [7]. A requirement of LIL3 for Chl and tocopherol synthesis was concluded from investigations using LIL3.1/LIL3.2 double mutants from *Arabidopsis thaliana*. Here, findings were based on a low yield of phytylated Chl a and a decrease in geranylgeranyl reductase (GGR) content. Data were interpreted to show a direct interaction between LIL3 and GGR [8]. Further mutagenesis studies reported a function of the LHC motif in LIL3 for anchoring GGR to the membrane and for dimerization of GGR [9]. However, in a yeast-two hybrid screen and by native PAGE analysis, LIL3 was found to directly interact with chlorophyll synthase (CHS), and protochlorophyllide oxidoreductase (POR). A direct interaction with GGR could not be verified [10]. Recently, binding of LIL3 to POR was corroborated [7].

In *Synechocystis*, HliD, a protein with partial homology to LIL3, was reported to form a complex with CHS, Ycf39 and YidC [11]. A complex isolated from *Synechocystis* was characterized to contain HliD, HliC, CHS, ß-carotene, and Chl a. The complex was reported to quench excitation energy and suggested to prevent a potential Chl mediated photo-damage of complexes regulating photosystem assembly or Chl synthesis [12]. Although data indicate a structural and functional participation of HliD/C or LIL3 in chlorophyll binding, no proof for direct binding of Chl has been provided. Here, we show selective binding of Chl a to *in vitro* expressed LIL3.

## Materials and methods

### Protein expression and purification

*Lil3* genes (TAIR, *A*. *Thaliana*, AT4G17600 and AT5G47110) were expressed in *E*. *Coli* BL21 (F–ompT hsdS(rB–mB–) gal dcm λ(DE3)) and harvested as described in [5]. LIL3 was purified by a modified method according to H. Paulsen for purification of LHCP [13]. In brief; Pellets were resuspended in 1 mL lysis buffer per 100 mL culture (50 mM TrisHCl, pH 8, 25% sucrose, 1 mM EDTA and 2 mg/200 μL lysozyme) and incubated 30 min at 4 °C. Lysates were vortexed vigorously prior to addition of 40 ng DNase 1, 10 mM MgCl_2_ and 1 mM MnCl_2_. The lysate was incubated for 30 min at RT prior to centrifugation at 10.000xg, 7 min at 4°C. For purification of *in vitro* expressed LIL3 protein, pellets were resuspended in 2 mL detergent buffer (200 mM NaCl, 1% (w/v) deoxycholic acid, 1% (v/v) Nonidet P40, 20 mM TrisHCl, pH 7.5, 2 mM EDTA and 10 mM β-mercaptoethanol) and were centrifuged at 10.000xg, 7 min at 4 °C. The pellets were resuspended in 2 mL Triton buffer (0.5% (v/v) Triton X-100, 20 mM TrisHCl, pH 7.5 and 1 mM β-mercaptoethanol) and were centrifuged at 10.000xg, 7 min at 4 °C. Pellets were washed with Triton buffer until pellets were easy to resuspend and almost white. Finally, the pellets were resuspended in resuspension buffer (50 mM Tris-HCl, pH 7.5, 1 mM EDTA and 10 mM β-mercaptoethanol) and were stored at -20 °C for reconstitution assays. The purified proteins were separated by SDS PAGE (12% (w/v)), stained with Coomassie Brilliant Blue (CBB) and were blotted with a His primary antibody as described in [5].

### Reconstitution of chlorophyll binding

Reconstitution assays were performed based on protocols as described [13, 14] in a reaction buffer containing 100 mM Tris, 5 mM 6-aminocaproic acid, 1 mM benzamidine and 12,5% sucrose, pH 11, and Chl a, a/b and b (Sigma, St. Louis, USA) in N-Dodecyl β-D-Maltoside (DDM) at 6 mM final concentration. In brief, 27 μM protein isolated from bacteria was centrifuged at 10.000 x g, 7 min and 4°C prior to resuspension in 10 μL reconstitution buffer with 100 mM DTT. Finally, pigments solubilized in DDM and reconstitution buffer (Chl a 6 μM and Chl b 6 μM) were added prior to heating samples to 100°C, 1 min followed by a two-hour incubation at RT in the dark.

### Isolation of reconstituted LIL3

LIL3 reconstituted with 6 μM Chl a/b in 6 mM DDM was separated on a HisTrap (GE Healthcare) using buffers containing N-Dodecyl β-D-Maltoside (DDM) at CMC. In Brief; Column was equilibriated with 10 column volumes of equilibration buffer (50 mM HEPES pH7.5, 250 mM NaCl, 25 mM imidazole, 0.2 mM DDM), sample applied and eluted with 250 mM imidazole (50 mM HEPES pH7.5, 250 mM NaCl, 250 mM imidazole, 0.2 mM DDM), fractions analyzed by LDS-Native (LN) PAGE on 3–12% polyacrylamide gels (Novex, Life technologies, California USA) with a cathode buffer supplemented with 74 μM LDS (not shown) and selected fractions applied to DLS measurements.

### Native PAGE

LIL3 bound to Chl was isolated by LDS-Native (LN) PAGE on 3–12% polyacrylamide gels (Novex, Life technologies, California USA) with a cathode buffer supplemented with 74 μM LDS [6]. Pigment and pigment binding protein were detected before and after protein staining by fluorescence scanning at 700 and 800 nm in a LI-COR Odyssey^®^ CLx and 633 nm in a Typhoon scanner (GE Healthcare, Buckingham, GB) using CBB as described [5].

### Microscale thermophoresis, MST

The intrinsic fluorescence of Chl a/b, was monitored at a final concentration of 30 nM Chl diluted in reconstitution buffer containing 6 mM DDM, while non-fluorescent LIL3.2 was titrated in a 1:1 dilution series (concentrations between 20.000 and 0.63 nm) in reconstitution buffer containing 6 mM DDM. After a two hour incubation at RT, samples were loaded into Monolith^™^ NT.115 MST Premium Coated Capillaries (NanoTemper Technologies, München, Germany) and measured using a Monolith NT.115 and MO.Control Software at RT, LED/excitation 20%, MST power setting 40%. Finally, a kinetic series for the Chl a LIL3.2 interaction was loaded into Monolith^™^ NT.115 MST Premium Coated Capillaries (NanoTemper Technologies, München, Germany) and sealed with acryl before repetitive measurements over a 50–150 min time scale using a Monolith NT.115 and MO.Control Software at RT, LED/excitation 20%, MST power setting 40%. Results are recorded as normalized fluorescence (F_norm_ = F_hot_/F_initial_) and presented as differential fluorescence (ΔF_norm_ = F_norm_ (bound)—F_norm_ (unbound) which reads as ΔF_norm_ = baseline corrected F_norm_ [‰].

### Dynamic light scattering, DLS

The solubilization of LIL3 (17.5 nM) in DDM was monitored by dynamic light scattering (DLS) at 25 °C in a N-Dodecyl β-D-Maltoside (DDM) concentration gradient ranging from 0–2 mM DDM using a quartz cuvette in the Zetasizer Nano ZSP instrument (Malvern, UK). The size of the 2 mM DDM detergent micelles was compared to 2 mM DDM micelles containing LIL3, Chl or affinity purified reconstituted LIL3/Chl a. The average value of the peak, weighted by the Y-axis parameter (mean hydrodynamic diameter) is given for all samples. Cumulative analysis is not valid for the polydisperse samples measured. Therefore, the distribution means in the linear range is reported, not Z-Average and PDI [15–19].

## Results

### Chlorophyll binding to LIL3 can be reconstituted

**Fig 1:**
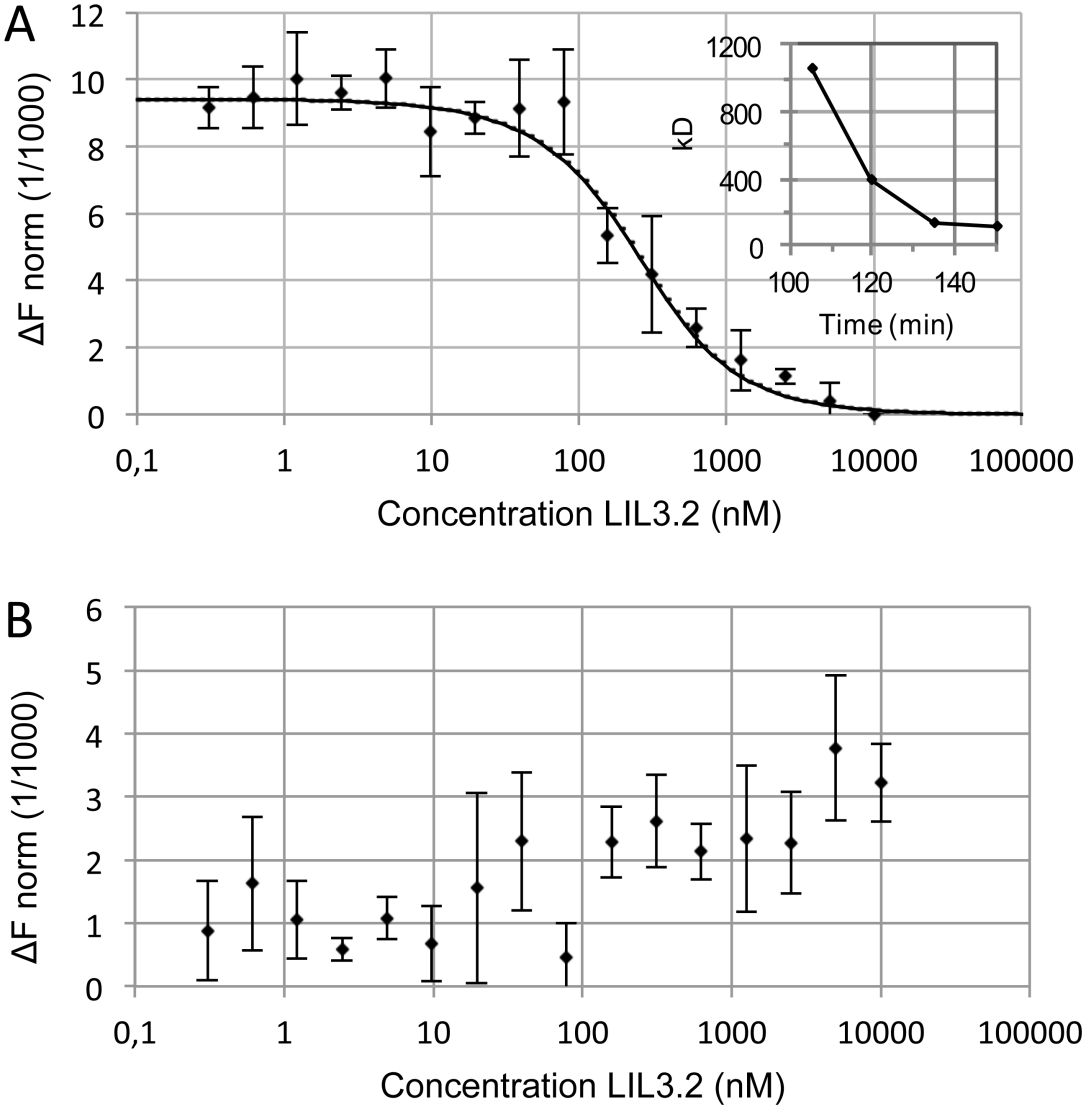
Dissociation constant and kinetics for Chl a binding to LIL3. Lil3 was solubilized at increasing concentrations 0.305 nM– 10 μM in the presence of a constant concentration (120 nM) of Chl in DDM micelles (6 mM). Normalized fluorescence difference from three MST measurements was plotted against the LIL3.2 concentrations (A). The time course for binding of Chl a was investigated by determination of Kd values upon initiation of reconstitution assays. Determined stable Kd values were plotted against the delay time after reaction onset (Inlet). The fluorescence difference averages, from three independent reconstitution assays of LIL3.2 with Chl a (A) and Chl b (B) whereby both reconstitutions were measured at 120 nM Chl after an incubation time of 2 hours.

The interaction of Chl with expressed and purified LIL3 was investigated in dodecylmaltoside (DDM) micelles using a process in which a temperature gradient induces a mass transport (thermophoresis). Typically, thermophoresis of molecules along a microscale temperature gradient is affected by molecular changes like size, charge or hydration shell, and a fluorescent reporter can be used to quantify a molecular interaction [20]. Here, Chl a (120 nM) was used as reporter and its affinity for LIL3 (0.3−10^4^ nM) was tested upon solubilization of both molecules in DDM (6 mM). For reconstitution, the mixture of both micelles was heated and upon cooling to RT, the interaction of Chl a with increasing concentrations of LIL3 was investigated over a time-frame of 150 minutes using microscale thermophoresis (MST, Fig 1A inlet).

At time point zero, and during the first 90 min, no significant change in the fluorescence difference analysis was recorded; however, Chl specific fluorescence showed high variability when the LIL3 concentration was increased (S1 Fig). This variability indicated that the interaction between both reaction partners was variable. In contrast, error bars were strongly decreased after about 90 min incubation and significant changes in fluorescence difference analysis were determined (Fig 1A (inlet, Kd kinetic) and S1 Fig).

The LIL3 concentration dependent change in the fluorescence difference analysis continuously decreased with time and saturated at a LIL3 concentration of about 115 nM after 150 min (Fig 1A, inlet and S1 Fig). Data showed that thermophoresis of Chl containing micelles was high at low concentration of LIL3 micelles, and decreased with the increase in LIL3 concentration. Prolongation of preincubation resulted in a change in thermophoresis of Chl containing micelles, until a saturation was reached at a ratio of about 1:1 for Chl (120 nM) and LIL3 (115 nM) containing micelles (Fig 1A, inlet, S1 Table)). The decrease in thermophoresis of Chl a-micelles indicated a binding between Chl a and LIL3 containing micelles. It was therefore concluded that the LIL3 concentration that established the thermophoretic change of Chl a mobility after a preincubation for more than 90 min reflected the dissociation constant (Kd) for the interaction. After a two-hour dark incubation of the reconstitution assays, an average Kd value of 146 nM was determined at an about 1:1 ratio for Chl a and LIL3 in DDM micelles (Fig 1A). However, no interaction with Chl b could be determined under the same conditions using MST (Fig 1B). The interaction between Chl a and Chl b and LIL3 was then tested further using gel electrophoresis.

### LIL3 retains Chl binding during native PAGE

**Fig 2:**
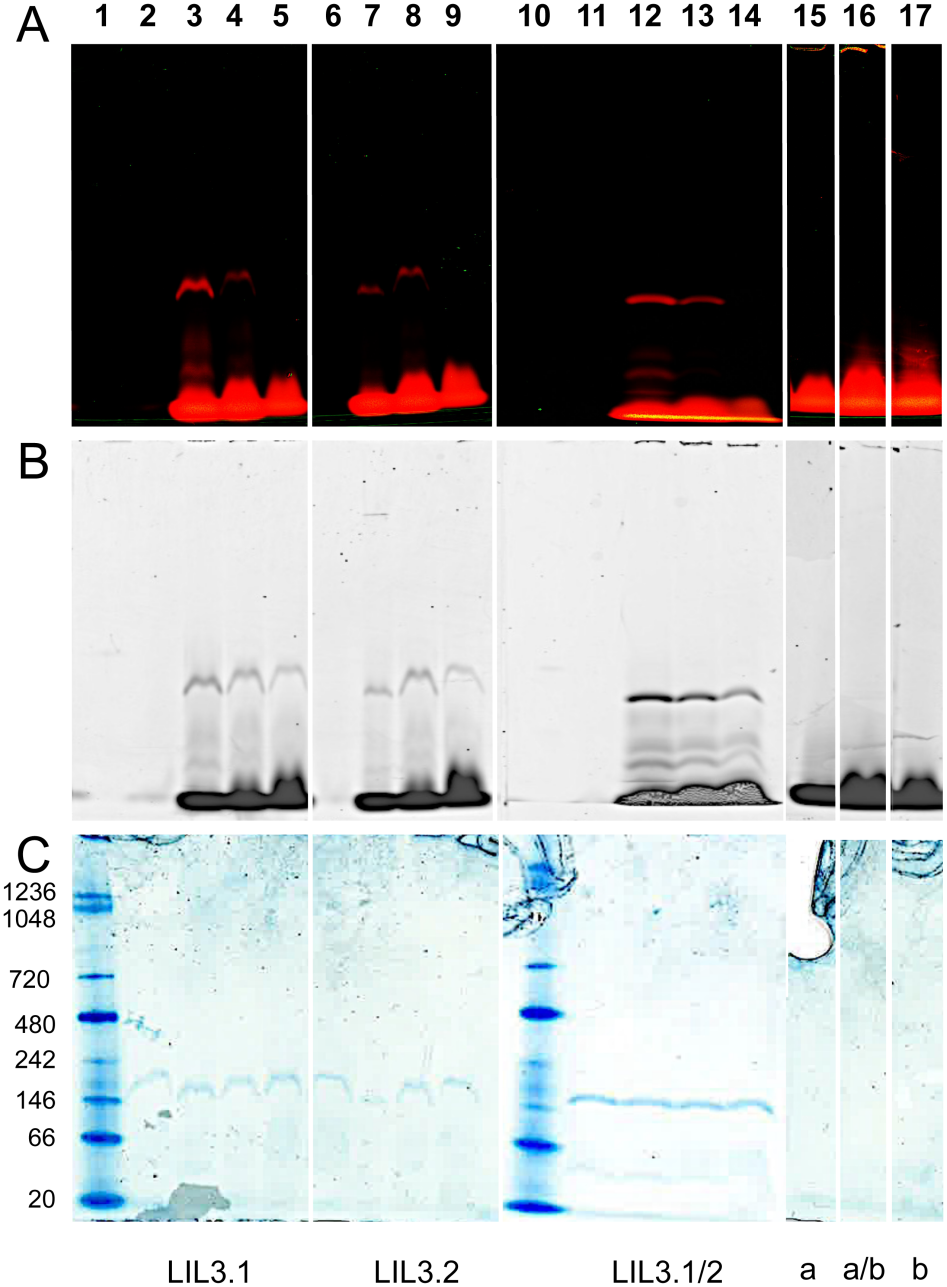
Electrophoretic mobility of chlorophyll and LIL3 upon reconstitution. The interaction of Chl a, Chl a/b and Chl b with LIL3 was visualized by electrophoretic mobility analysis using native gels. Recombinant LIL3 was reconstituted with Chl a, Chl a/b and Chl b, and assay components were isolated using native 3–12% LN-PAGE. Reconstitution assays were loaded on the basis of equal pigment concentration (A-C, lanes 1–17) and the relative mobility of components were compared to molecular weight standard proteins (C, kDa). The mobility of Chl a and Chl b in native gels was determined by laser excitation/emission scanning at 680/700 nm (A) and 633/670 (B). The mobility of LIL3 in the gels was determined by in-gel staining using colloidal Coomassie (C). Lane numbers refer to native mark (1), and reconstitution assays containing LIL3 (27 μM), Chl a or Chl b (6μM), Chl a/b (3μM Chl a and 3μM Chl b). LIL3 isoforms: LIL3.1 (lanes 2–5), LIL3.2 (lanes 6–9) and LIL3.1/2 (lanes 11–14). Lanes: LIL3.1 without Chl (2), plus Chl a (3), plus Chl a/b (4), plus Chl b (5), LIL3.2 without Chl (6), or plus Chl a (7), plus Chl a/b (8), plus Chl b (9), Native mark (10), LIL3.1/2 without Chl (11), plus Chl a (12), plus Chl a/b (13), plus Chl b (14). Reconstitutions without LIL3: Chl a (15), Chl a/b (16) and Chl b (17).

*In vitro* expressed LIL3.1, and LIL3.2, and a mixture of LIL3.1 and LIL3.2 (LIL3.1/2) (27 μM) were each reconstituted with either Chl a, Chl b, or a mixture of Chl a and b (Chl a/b) and isolated by native PAGE (Fig 2). The gel was scanned for Chl a fluorescence at an Ex/Em of 680/700 nm (Fig 2A) and for Chl a and Chl b at an Ex/Em of 633/670 nm (Fig 2B). To evaluate the relative mobility of reconstituted LIL3-Chl micelles against the mobility of LIL3 or Chl micelles, Chl was determined by fluorescence and LIL3 protein was stained with Coomassie Brilliant Blue (CBB) (Fig 2C). Fluorescence scans showed a fluorescent band in a low-mobility fraction at about 146 kDa for LIL3 reconstituted with Chl a, Chl a/b, and Chl b (Fig 2A and 2B, lanes 3–5, 7–9 and 12–14). Fluorescence was also recorded in a high-mobility fraction at a molecular weight of about 20 kDa for reconstitution assays and Chl micelles incubated in the absence of LIL3 (Fig 2A and 2B, lanes 3–5, 7–9, 12–14, and 15–17). It was therefore concluded that the band at 20 kDa corresponds to Chl micelles.

Coomassie staining of the LIL3 protein controls, incubated in the absence of Chl, showed a very similar mobility to reconstituted LIL3 at about 146 kDa; however, no fluorescence was recorded from the protein controls (Fig 2, lane 2, 6 and 11). The finding showed that LIL3 and LIL3 reconstituted with Chl could not be differentiated by electrophoretic mobility in the native gel. This indicated that micelles containing LIL3 are similar in hydrodynamic diameter to micelles containing reconstituted LIL3 (Fig 2C, lane 1–14). Fluorescence of the 146 kDa band corroborated an interaction between LIL3 and Chl a (Fig 2A, lane 3).

**Fig 3:**
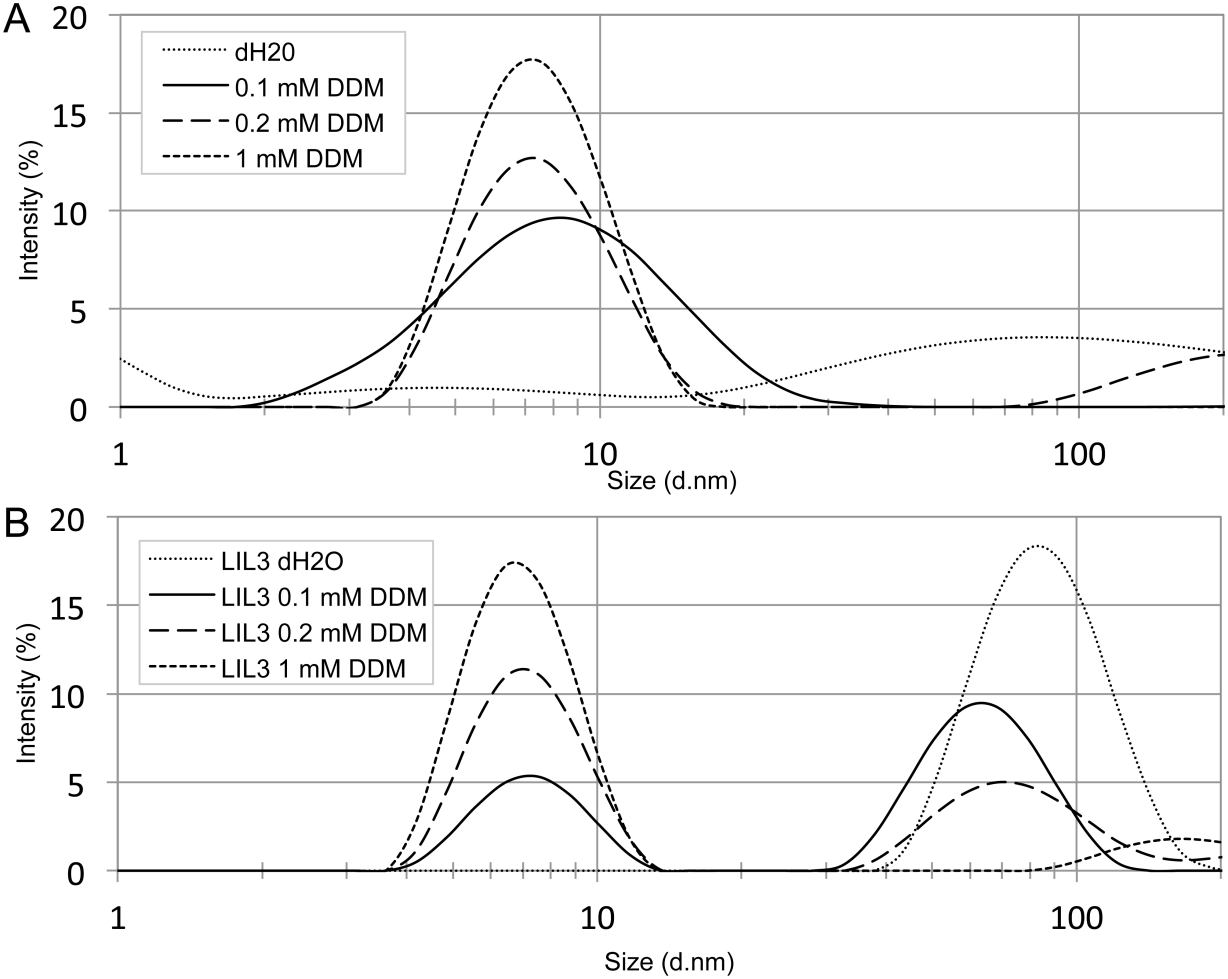
Intensity distribution of LIL3 upon solubilization in DDM micelles. Formation of DDM micelles (A) and of recombinant LIL3.2 (17.5 nM) (B) were monitored by dynamic light scattering upon solubilization using 0, 0.1, 0.2 and 1 mM DDM.

**Fig 4:**
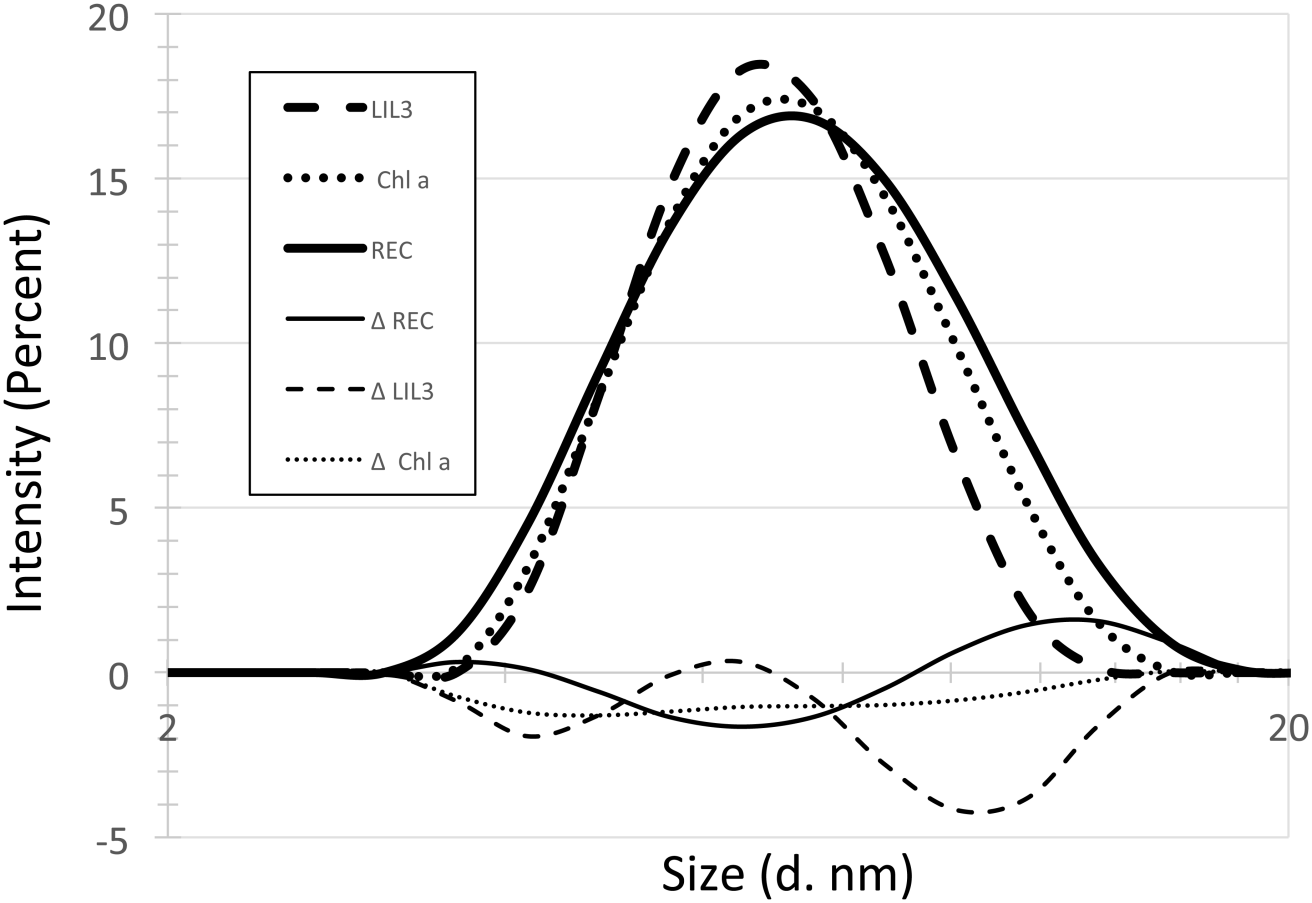
Intensity distribution of reconstituted Lil3 complexes at RT. LIL3.2 (LIL3), and Chlorophyll a (Chl a) were solubilized in 2 mM DDM micelles (DDM) and the solubilized reaction partners were reconstituted (REC). The intensity distribution profile (Diameter (d. nm)) of LIL3, Chl a, and of affinity purified LIL3.2 reconstituted with Chl a were determined. The difference spectra of the intensity distribution of reaction partners LIL3 (Δ LIL3), Chl a (Δ Chl a) and reconstituted LIL3 (Δ REC) against DDM (DDM subtracted) were determined.

Results showed no fluorescent band upon reconstitution with Chl b, indicating that LIL3 did not interact with Chl b (Fig 2A, lane 5). However, when the sensitivity for Chl b was increased by laser excitation at 633 nm, increased fluorescence was recorded indicating an interaction of Chl b with LIL3. Quantification of fluorescence yield from native gel bands, showed a 5-fold decrease for Chl b relative to Chl a interaction (Fig 2B, lanes 3 and 5). Controls containing Chl a and b in a 1:1 ratio showed that the yield of fluorescence from the LIL3 band decreased and that the yield of LIL3.1 for binding Chl a was decreased about 50% when half of Chl a was replaced by Chl b indicating that the reconstitution yield was dependent on the concentration of Chl a present in the reconstitution assay (Fig 2A and 2B, lanes 3 and 4). However, the yield of reconstituted LIL3.2 remained about constant under the same experimental conditions indicating that LIL3.2 had a higher affinity for binding Chl a (Fig 2A and 2B, lanes 3/4 and 7/8). We noted that reconstitutions performed in the presence of both LIL3 isoforms, LIL3.1 and LIL3.2, resulted in the most stable separation of complexes in bands, a fluorescence yield dependent on the concentration of Chl a, and a clear preference for binding of Chl a (Fig 2A and 2B, lanes 12–14). Nevertheless, native PAGE analysis indicated an interaction of LIL3 with Chl b and that the particle diameter of LIL3 containing micelles was not changed upon binding of Chl to LIL3. We therefore investigated the micellar diameter directly by dynamic light scattering (Figs 3 and 4).

### Changes in the distribution of micelles containing Lil3

In dynamic light scattering analysis (DLS), changes in the mean hydrodynamic diameter and the poly-dispersity of DDM micelles were determined from the intensity distribution of particles with characteristic Brownian motion [21]. The mean particle diameter of DDM micelles (2 mM) (Fig 3A) and of micelles containing reconstituted and affinity purified LIL3 complexes (Fig 3B) was determined. When LIL3.2 (17.5 nM) was dissolved in water, DLS analysis showed an intensity distribution mean diameter of 91.3 nm (Fig 3B). Upon solubilization in DDM, the mean intensity of the distribution profile decreased showing that aggregation of LIL3 was dissolved. In parallel with an increase in the DDM concentration from 0.1, to 0.2 and 1 mM DDM, the concentration of LIL3-DDM micelles with an intensity distribution mean diameter of 6.5 nm was increasing (Fig 3B).

DDM micelles showed a distribution means of 7.53 nm relative to the 6.5 nm observed when LIL3 was solubilized by DDM. DLS analysis of both experiments shows that both distribution profiles overlap; however, while the intensity of both distribution profiles is maintained, solubilized LIL3 shows a sharper and less disperse distribution with a relative decrease in the means. Difference analysis (S2A and S2B Fig) revealed a higher concentration of DDM micelles with small (4.2 nm) and large hydrodynamic diameter (11.7 nm) relative to solubilized LIL3. This shows that solubilization of LIL3 by DDM is well determined by the decrease of small and large DDM micelles via the distribution profile analysis (S2 Fig) as well as by the decrease of the intensity of the distribution profile of LIL3 aggregates at about 79 nm (Fig 3A and 3B).

Solubilization of Chl a in 2 mM DDM hardly affected the mean diameter of DDM micelles; whereas, solubilization of reconstituted LIL3 resulted in a small increase of the distribution means corresponding to an asymmetric change of the distribution profile (Fig 4). DLS analysis showed that the distribution means were dominated by the high concentration of DDM micelles (1–2 mM DDM). The concentration of solubilized reaction partners Chl (30 nM) and LIL3 (20 nM) was small. Nevertheless, changes in the distribution profiles were well detectable using difference analysis.

Difference spectra between reconstituted LIL3 and DDM micelles (Fig 4, ΔREC–DDM) showed that for solubilization of reconstituted LIL3, the contribution of DDM micelles with a mean diameter around 6.5 nm were decreased indicating that the distribution profile of the DDM micelles were changing upon solubilization of reconstituted LIL3. In contrast, changes in the distribution profile of reconstituted LIL3 particles relative to DDM micelles showed a mean diameter intensity at about 13.5 nm (Fig 4). This indicated that the purified reconstituted LIL3 accumulated DDM in the presence of the DDM micelles. Also solubilization of LIL3 aggregates indicated that DDM binding to the LIL3 monomers increased the mean LIL3 diameter (Fig 4). In addition, data showed that the distribution profiles of DDM micelles were hardly changed upon solubilization of Chl a, Chl a/b and Chl b (Fig 4, and S3 Fig). We concluded that the increased intensity distribution at about 13.5 nm could be based on an increased affinity of reconstituted LIL3 (6.5 nm), for binding DDM (6.5–7.5 nm) (Fig 4). Hence, data may indicate that binding of Chl upon reconstitution of LIL3 alters the structure of LIL3 and its hydrodynamic diameter in the presence of DDM micelles.

## Discussion

We show by mobility analysis of micelles that Chl a binding to LIL3 can be reconstituted *in vitro* in the absence of carotenoids, lipids and of Chl b. A protocol for *in vitro* reconstitution of Chl binding to LIL3 is presented. The method has been established on the basis of protocols developed by Plumley and Schmidt [14] and Paulsen [13] for Chl binding to LHCP. The binding of Chl to LIL3 is characterized by MST, DLS, and native PAGE. All three methods investigate changes in the mobility of solubilized LIL3 micelles in the presence and absence of an interaction with Chl micelles. Selective binding of Chl a to LIL3 is demonstrated using MST.

### Dynamic light scattering reveals structural transformations upon solubilization and reconstitution of LIL3

The determined size of DDM micelles corresponds well to literature [22–24]. However, the mean diameter of DDM micelles (7.53 nm) decreased in the presence of LIL3 (6.5 nm). At concentrations below or close to CMC (0.17 nM), a mixture of DDM organized in micelles and single DDM molecules are present which have different structures, mass and rotational volume resulting in a polydisperse distribution profile (Fig 3A, x-axis). Above the CMC (1 mM), the majority of the DDM is present as micelles and hence lower poly-dispersity is observed. When LIL3 is solubilized in DDM (Fig 3B) the mass of the micelle is increased and the rotational volume affected by the more disc like structure. As the DDM concentration is increased, increasing amounts of LIL3 are solubilized. However, while dispersity is not affected as compared to DDM micelles (Fig 3B, x-axis), intensity increases (Fig 3A, y-axis). Thus, indicating that the rotational volume, mass and structural organization are not changing, only the amount of solubilized LIL3 increases. Therefore, the apparent decrease in the distribution means observed for solubilized LIL3 likely reflects the microstructural transformation of DDM micelles. Similar microstructural transformations for micelle mixtures were reported upon varying the charges in aqueous [C_8_SO_4_]/[CTAB] [25].

### Characterization of LIL3 interaction with Chl using DLS and native PAGE

Mobility analysis of reconstituted LIL3 by DLS and native PAGE indicated that chlorophyll is bound to LIL3. However, neither of the methods differentiated between a binding of Chl a and Chl b. In contrast, thermophoretic mobility analysis showed that LIL3 interacted only with Chl a (Fig 1). Finally, mobility analysis by native PAGE did not differentiate between LIL3 and reconstituted LIL3 bands (Fig 2).

How can these findings be explained? In DLS, changes in the mean hydrodynamic diameter of dissolved molecules can be determined [21, 26]. Reconstituted LIL3 micelles peaked at 13.5 nm in differential analysis of the distribution means (Fig 4) indicating that reconstitution increased the hydrodynamic diameter relative to micelles containing Chl or LIL3 by about 7 nm (Fig 4). In addition, the electric field in the native PAGE analysis gave the corresponding result. An increased mobility of micelles containing only Chl relative to reconstituted LIL3 (Fig 2) indicated a smaller hydrodynamic diameter.

A higher charge density cannot explain the effect observed. LIL3.1 and LIL3.2 micelles contain 7.8 and 6.8 negative charges at pH 7, respectively; whereas, micelles containing Chl are uncharged. Micelles containing LIL3 were therefore retarded based on a larger hydrodynamic diameter despite a higher charge density. DLS analysis had also shown that LIL3 aggregated in the absence of detergent (Fig 3) [27]. This indicates that the larger hydrodynamic diameter of LIL3 could have resulted from LIL3 aggregation during native PAGE. Specifically, aggregation could have taken place when the sample is concentrated in bands during the gel entry phase. Here, micelles get charged by charged detergents present in the cathode buffer tank. In the electric field, charged micelles with a small hydrodynamic diameter follow the buffer front, and are separated from the larger LIL3 containing micelles by the gel-filtration process. Since the volume of a native gel is typically detergent free, the stability of detergent binding to proteins is compromised [28]. This indicates that LIL3 specific mobility in native PAGE could result from an aggregation of LIL3 whereby a weak interaction between LIL3 and Chl b is fixed. We conclude that our mobility studies using DLS and native PAGE are not sufficient to prove an interaction between Chl and LIL3; whereas thermophoresis provides a specific binding affinity for Chl a to LIL3 [20, 29–31].

### Chl binding to protein

In thermophoresis, highly variable chlorophyll specific fluorescence is determined when the interaction of LIL3 and Chl was investigated in the absence of a pre-incubation at time point zero. Variability was especially noted when the ratio of Chl:LIL3 decreased (S1 Fig). This may indicate that the interaction between chlorophyll and LIL3 is not stable at that point. In contrast, after a pre-incubation period of 90 min, the binding state improved and Chl based fluorescence variability was strongly decreased corroborating an improved stability of the interaction between Chl and LIL3 (S1 Table and Fig 1). Thermophoresis remains nevertheless an indirect method that monitors by fluorescence how the mobility of Chl micelles changes upon interaction with LIL3 micelles.

Direct biochemical evidence for binding of bacteriochlorophyll (BChl) was first shown by crystallization of the bacterial reaction center of photosystem II from *Rhodopseudomonas viridis* [32]. Structural data of the Chl binding sites show that the Chl’s non-polar parts of the tetrapyrrol and the phytyl side chain and the proteins non-polar hydrophobic amino acids resemble non-polar phases for interaction between Chl, protein and lipids [32, 33]. Polar amino acid side groups and the peptide backbone are used for binding Chl [33]. In LIL3.1, amino acids E170, N173 and R175 and in Lil3.2 amino acids E173, N176 and R178 have been characterized as potential candidates for interaction with Chl a (AA numbers refer to the proteins full length sequence) [5]. The oxygen and nitrogen atoms of polar amino acid side groups, (Glu, Asp, Arg and His) can operate as Lewis acids for coordination of the central Mg2+ ion in Chl [32–35], and prevent electrostatic formation of anions during light driven electron transfer [32]. Cooperation with polar amino acids at positions i+/-3 and i+/-4 along alpha-helical side groups with the carbonyl-oxygens of the 13^1^—keto, or 132—keto-methoxy tetrapyrrol side groups, or with the 17^3^—ester carbonyl group can arrest rotation of Chl around the coordinated Mg^2+^ [34–36].

In LIL3.1 and LIL3.2, AA pairs E170, N173, and E173, N176 fulfil the criteria for positional binding of Chl a; also, AAs pairs E170, R175, and E173, R178 fulfil the criteria for coordination of the central Mg^2+^ ion and for formation of salt bridges in a homo- and hetero-dimer [5]. In general, also amino acids containing aromatic side chains like Phe, Tyr and Trp, are involved to position the tetrapyrrol, whereby the special pair of Chl is bound to bacteriopheophytin via hydrogen bond interaction, and accessory Chl’s are associated through van der Waals interactions only [32]. Additional interaction between the non-polar parts of Chl with non-polar phases of the protein and the lipids can finally orient the Chl molecule relative to the protein surface, the lipid phase and positions Chl in 3D at its binding site [37]. Chl molecules are typically bound by interaction with amino acid residues distant in primary sequence [33, 38]. This emphasizes the need to investigate the full LIL3 sequence to elucidate interaction with Chl a and how binding of Chl b is sorted out.

### LIL3 is central for regulation of Chl biosynthesis and its delivery

Isolation of fluorescent native complexes upon de-etiolation of barley identified co-migration of LIL3 with CHS, POR and GGR. All proteins, except LIL3, had been described to be of central importance for the regulation of Chl synthesis. However, these proteins had not been identified in interaction with Chl. This indicated a structural and/or functional significance for binding of Chl to LIL3 [5, 10]. Several investigations suggested that LIL3 and its homologs are involved in chlorophyll biosynthesis and delivery of Chl to the protein complexes of the photosynthetic machinery. LIL3 was found to directly interact with chlorophyll synthase (CHS) and POR [10]. Hey et al, recently verified the LIL3 POR interaction [7, 10]. MST analysis provides a dissociation constant for Chl a binding to LIL3 upon complete solubilization of each reaction partner in DDM. In a recent work concerning LIL3 tryptophane quenching was used to show an interaction of pigments with LIL3 [7],. The study was conducted in the absence of a solubilization of LIL3 and therefore likely represents the interaction of pigment with trypophane in protein aggregates [5, 7]. An interaction of LIL3 and also of its cyanobacterial homolog HliD/C with CHS supports the hypothesis that Chl binding to LIL3 is central for regulation of Chl biosynthesis and photosystem assembly [7, 10, 11].

LIL3 has been described to stabilize and anchor GGR to the membrane via the LHC motif [8]. Although this finding claimed a direct interaction between GGR and LIL3, it could not be verified opening for the possibility of an indirect interaction between LIL3 and GGR [8, 10, 39]. Recently, a protective function was suggested based on energy transfer measurements for the LIL3 homolog HliD; whereby carotenoid and Chl molecules bound to HilD where proposed to protect protein complexes during Chl biosynthesis and photosystem assembly [12]. At present, we can´t exclude that carotenoids bind to LIL3 *in vivo*; however, we do not find evidence that carotenoids are required for binding Chl a to LIL3 *in vitro*. The *in-vitro* reconstitution protocol makes it now possible to investigate the Chl a binding site in LIL3 using genetic screens. It may also be investigated whether the incubation period of more than 90 min was required to saturate Chl binding sites *in vitro* or reflected the lack of assembly factors in the *in vitro* approach.

## Supporting information

S1 Fig MST measurement of LIL3.2 Chl a interaction without pre-incubation of reaction partners. Lil3 was solubilized at increasing concentrations 0.305 nM –10 μM in the presence of a constant concentration (120 nM) of Chl in DDM micelles (6 mM). Normalized fluorescence difference from three independent MST measurements at time point zero was plotted against the LIL3.2 concentrations (A). The time course for binding of Chl a was investigated by determination of Kd values upon initiation of reconstitution assays. Determined stable Kd values were plotted against the delay time after reaction onset (S1 Table).
(TIF)

S2 Fig Difference analysis of intensity distribution profiles between DDM micelles and solubilized Lil3. The distribution profile of DDM micelles (A, 1 mM DDM) and of solubilized LIL3.2 (B, LIL3 in 1 mM DDM) was analyzed. Difference analysis (A–B) shows how the intensity (Intensity (%) of the DDM micelle distribution profile (Diameter (d. nm)) changes upon solubilization of LIL3.
(TIF)

S3 Fig LIL3 associates with Chlorophyll in DDM micelles. Recombinant LIL3.2 bound to Chl a (A), Chl a/b (B) and Chl b (C) was affinity purified at CMC (0.2 mM DDM) and analysed by dynamic light scattering at 25 °C in 2 mM DDM (LIL3 Chl a, LIL3 Chl a/b and LIL3 Chl b). As controls, the dynamic light scattering of LIL3 in 2 mM DDM (LIL3), Chl in 2 mM DDM (Chl a, Chl a/b and Chl b) and the micelle in a concentration of 2 mM DDM (DDM) were measured.
(TIF)

S1 Table Dissociation constant and kinetics for Chl a binding to LIL3. Lil3 was solubilized at increasing concentrations (0.305 nM– 10 μM) in the presence of a constant concentration of Chl (120 nM) in DDM micelles (6 mM). The time course for binding of Chl a was investigated by determination of Kd values upon initiation of reconstitution assays. Determined stable Kd values were plotted against the delay time after reaction onset.
(TIF)
