# Supplementary figures and images for: Characterization of chlorophyll binding to LIL3
Source: PLoS One. 2018 Feb 1;13(2):e0192228. doi: 10.1371/journal.pone.0192228 (PMC5794176; doi:10.1371/journal.pone.0192228)

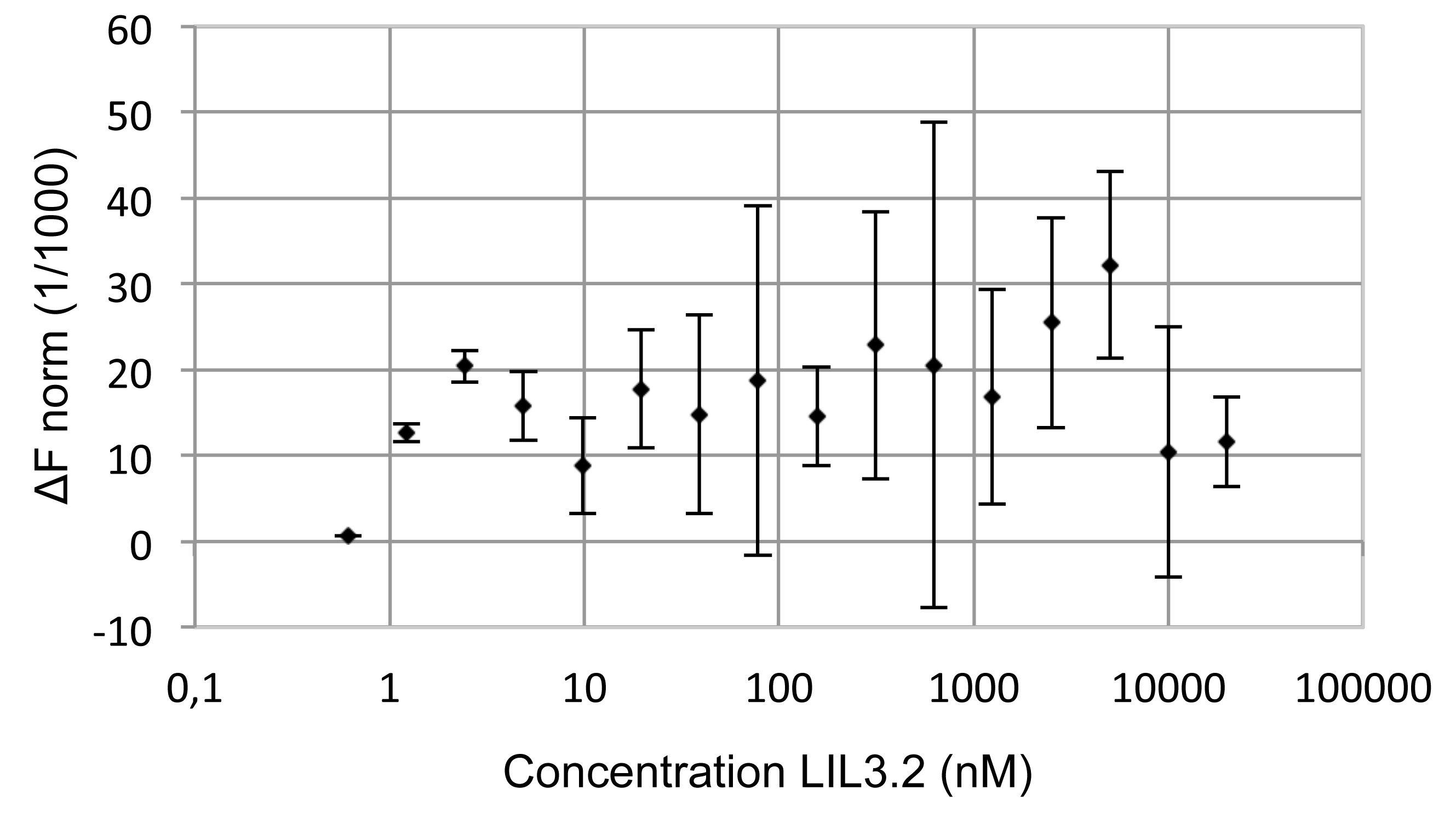

Supplement: S1 Fig — Lil3 was solubilized at increasing concentrations 0.305 nM –10 μM in the presence of a constant concentration (120 nM) of Chl in DDM micelles (6 mM). Normalized fluorescence difference from three independent MST measurements at time point zero was plotted against the LIL3.2 concentrations (A). The time course for binding of Chl a was investigated by determination of Kd values upon initiation of reconstitution assays. Determined stable Kd values were plotted against the delay time after reaction onset (S1 Table). (TIF) [file pone.0192228.s001.tif]

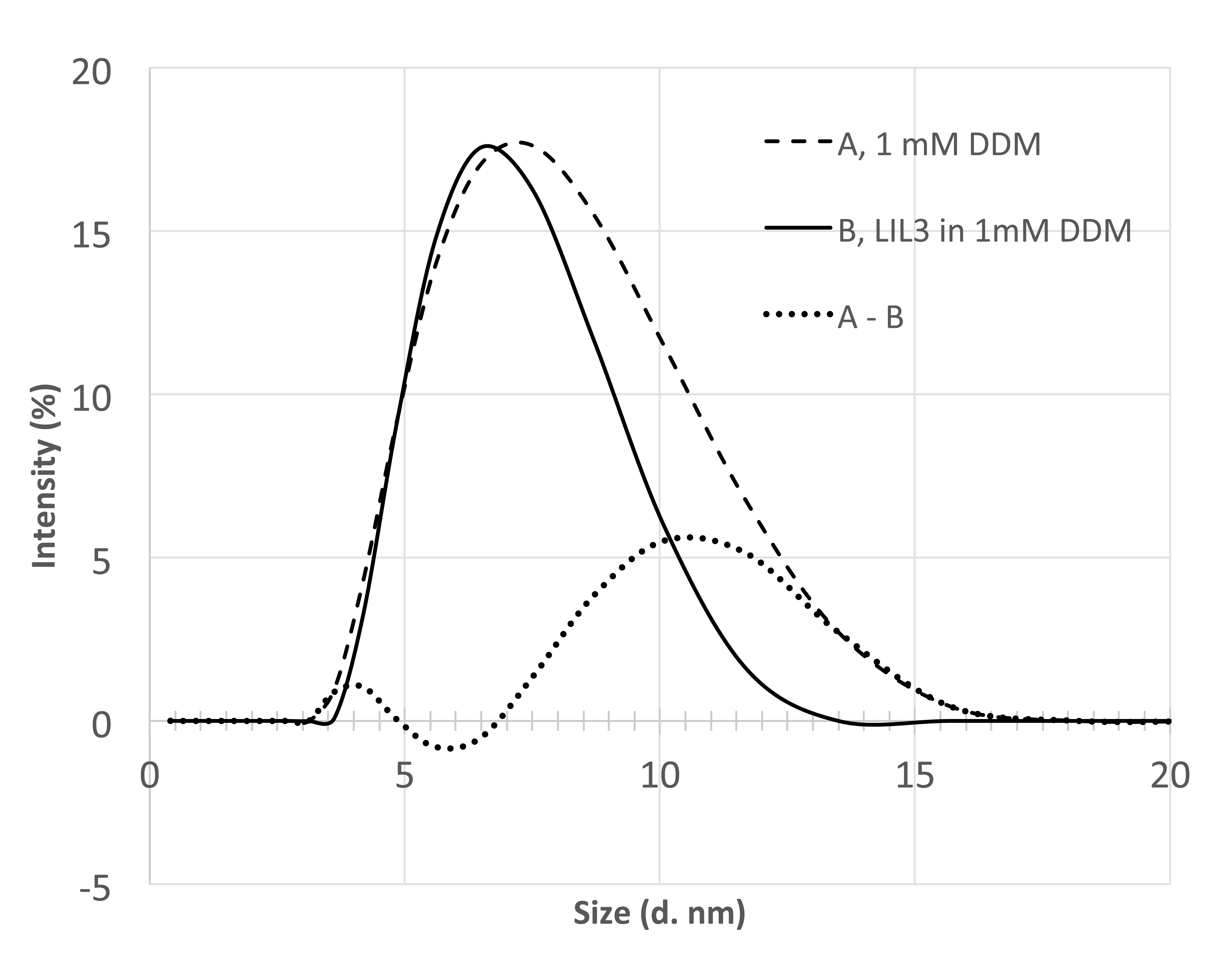

Supplement: S2 Fig — The distribution profile of DDM micelles (A, 1 mM DDM) and of solubilized LIL3.2 (B, LIL3 in 1 mM DDM) was analyzed. Difference analysis (A–B) shows how the intensity (Intensity (%) of the DDM micelle distribution profile (Diameter (d. nm)) changes upon solubilization of LIL3. (TIF) [file pone.0192228.s002.tif]

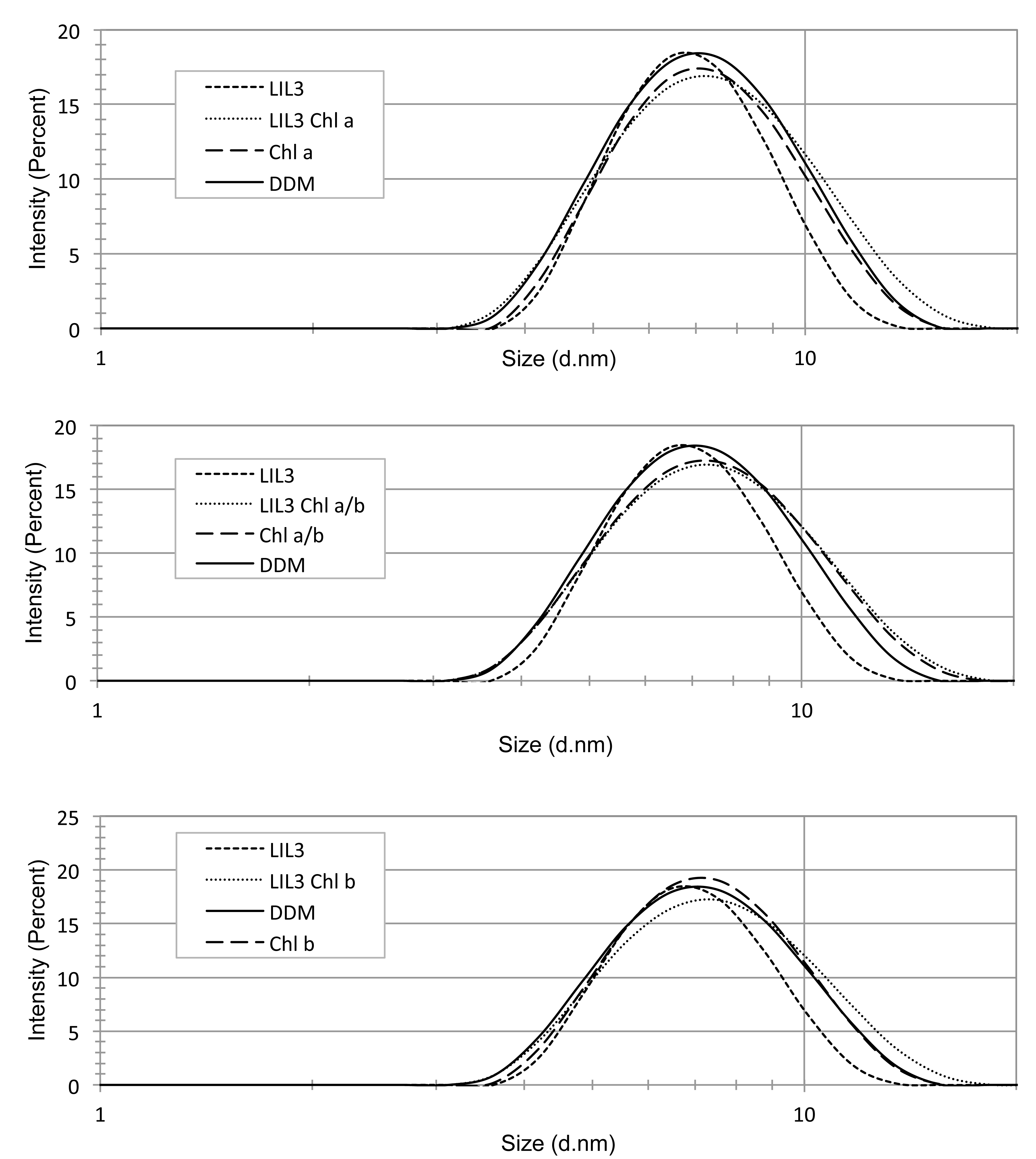

Supplement: S3 Fig — Recombinant LIL3.2 bound to Chl a (A), Chl a/b (B) and Chl b (C) was affinity purified at CMC (0.2 mM DDM) and analysed by dynamic light scattering at 25 °C in 2 mM DDM (LIL3 Chl a, LIL3 Chl a/b and LIL3 Chl b). As controls, the dynamic light scattering of LIL3 in 2 mM DDM (LIL3), Chl in 2 mM DDM (Chl a, Chl a/b and Chl b) and the micelle in a concentration of 2 mM DDM (DDM) were measured. (TIF) [file pone.0192228.s003.tif]

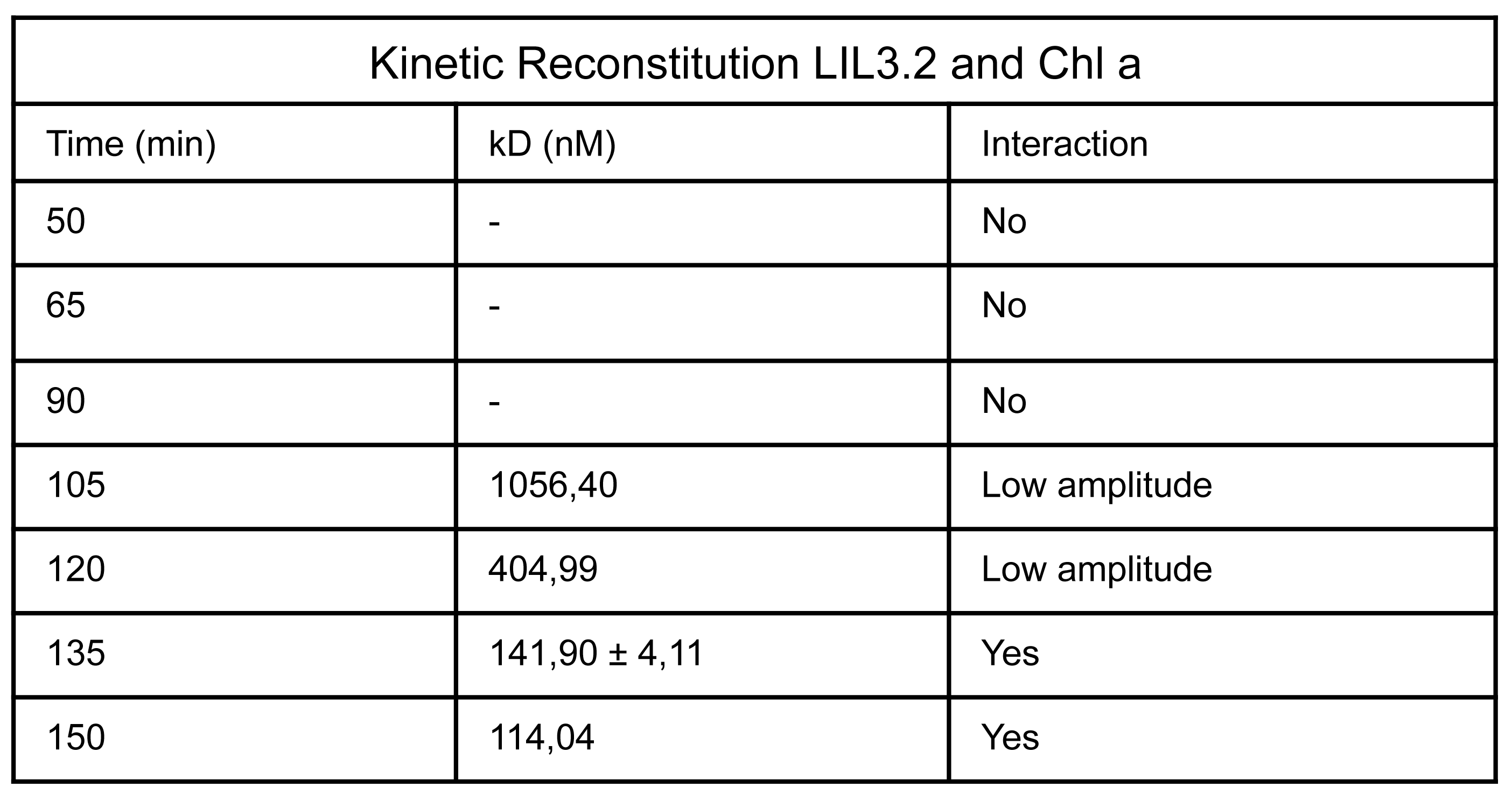

Supplement: S1 Table — Lil3 was solubilized at increasing concentrations (0.305 nM– 10 μM) in the presence of a constant concentration of Chl (120 nM) in DDM micelles (6 mM). The time course for binding of Chl a was investigated by determination of Kd values upon initiation of reconstitution assays. Determined stable Kd values were plotted against the delay time after reaction onset. (TIF) [file pone.0192228.s004.tif]
